# Dynamic Perviousness Predicts Revascularization Success in Acute Ischemic Stroke

**DOI:** 10.3390/diagnostics14050535

**Published:** 2024-03-03

**Authors:** Gergely Bertalan, Roxane Duparc, Miklos Krepuska, Daniel Toth, Jawid Madjidyar, Patrick Thurner, Tilman Schubert, Zsolt Kulcsar

**Affiliations:** Department of Neuroradiology, University Hospital Zürich, Frauenklinikstrasse 10, 8091 Zürich, Switzerlandmiklos.krepuska@usz.ch (M.K.); daniel.toth@meduniwien.ac.at (D.T.); jawid.madjidyar@usz.ch (J.M.); patrick.thurner@usz.ch (P.T.); tilman.schubert@usz.ch (T.S.); zsolt.kulcsar@usz.ch (Z.K.)

**Keywords:** acute ischemic stroke, mechanical thrombectomy, dynamic perviousness of thrombi, computed tomography

## Abstract

Background: The predictive value of thrombus perviousness in acute ischemic stroke (AIS), as measured by computed tomography (CT), has been intensively studied with conflicting results. In this study, we investigate the predictive potential of the novel concept of dynamic perviousness using three-dimensional (3D) volumetric evaluation of occlusive thrombi. Methods: The full thrombus volume in 65 patients with a hyperdense artery sign on non-contrast CT (NCCT), who underwent mechanical thrombectomy (MT), was segmented. Perviousness maps were computed voxel-wise for the entire thrombus volume as thrombus attenuation increase (TAI) between NCCT and CT angiography (CTA) as well as between CTA and late venous phase CT (CTV). Perviousness was analyzed for its association with NIHSS at admission, Thrombolysis In Cerebral Infarction (TICI) score, and number of MT passes. Results: The mean late-uptake TAI of thrombi with NIHSS scores greater than 21 at admission was approximately 100% higher than for lower scored NIHSS (*p* between 0.05 and 0.005). Concerning revascularization results, thrombi requiring less than four MT passes had ca. 80% higher group mean late-uptake TAI than clots requiring four or more passes (*p* = 0.03), and thrombi with TICI score III had ca. 95% higher group mean late-uptake TAI than thrombi with TICI II (*p* = 0.03). Standard perviousness showed no significant correlation with MT results. Conclusion: Standard thrombus perviousness of 3D clot volume is not associated with revascularization results in AIS. In contrast, dynamic perviousness assessed with a voxel-wise characterization of 3D thrombi volume may be a better predictor of MT outcomes than standard perviousness.

## 1. Introduction

Acute ischemic stroke (AIS) is one of the most frequent causes of death and an important reason for long-term disability among people over 60 years of age [1]. Over 85% of all strokes are of ischemic origin where a blood clot obstructs a cerebral blood vessel and interrupts perfusion to brain tissue [2]. Mechanical thrombectomy (MT), combined or not with systemic thrombolysis, became the gold standard for AIS treatment with large vessel occlusion [1]. In this endovascular procedure, the thrombus is removed using catheterization techniques, with a special device called a stent-retriever or via aspiration with a large bore catheter. The goal of treatment is fast and complete revascularization [2], which is associated with good clinical outcomes and lower mortality [3,4]. However, in approximately 10–20% of MT, substantial revascularization is not achieved [5,6,7,8,9], resulting in unsatisfactory clinical outcomes after the intervention. Therefore, efforts have been made to predict thrombus properties from pre-treatment imaging, as such, predictions could accelerate decision-making before recanalization and improve clinical outcomes.

The thrombus properties and composition are important factors for determining revascularization success and thus good clinical outcome. In vitro analyses have shown that certain devices successfully extract certain thrombus types, but not others [10,11]. For example, aspiration is more successful with soft clots, whereas extraction of harder clots should be attempted with stent-retrievers [10,11]. Thrombus length [12,13], occlusion location [8,14], vessel tortuosity [15], histological composition [10,16,17], perviousness [18,19], stiffness [20], and thrombus age [21] have all been associated with treatment success and outcome in various studies [22].

Neuroimaging is the cornerstone for the diagnosis and management of AIS, by providing information on the salvageable and infarcted brain tissue, vessel occlusion, thrombus location [19], perviousness [23], length [24], volume [25], shape [26], and composition [22,27]. All of these factors will define revascularization attempts and will help to select the tools and technique of MT. Computed tomography (CT) is present in most emergency departments and is widely used to make time-critical decisions due to the fast acquisition times. Due to the concept of “time is brain”, the requirements of imaging diagnostics are to give the correct diagnosis of AIS, evaluate the damaged and yet salvageable brain tissue, and depict the vessel occlusion and all of these in the shortest time possible. In depth, time-dependent imaging analysis of occlusive thrombus structure would not be ethical, thus we need to rely on the data that is provided by the routine scans. On non-contrast CT (NCCT) images, clots may show up as a hyper-attenuated area (the hyperdense artery sign). Its presence is associated with a higher content of red blood cells [23,28,29] and it is often absent in platelet-rich thrombi [29]. On CT angiography (CTA), the blood vessels are contrast-enhanced and the occluding, impermeable thrombus is visible as a lack of signal. With a CTA clot burden, collateral circulation, thrombus perviousness, and clot length if distal perfusion is sufficient can be assessed [30].

Several CT image features have been reported to be associated with MT outcome but findings are controversial with different studies showing opposite results. Many studies focused on CT signal intensity and the presence of a hyperdense artery sign on NCCT. Shin et al. observed that red-blood-cell-rich clots were associated with the presence of a hyperdense artery sign and with successful recanalization [16]. Similarly, clots from patients with a good angiographic outcome had higher mean CT density than the ones from those with poor outcomes [31]. However, several studies reported contradictory findings [23,32,33]. According to their observations, higher HU values were associated with higher fibrin content, not red blood cells, and with longer intervention times [33], or showed no correlation with recanalization success [23,32], nor red blood cell content [23]. Thus, the connection between the clot imaging features on standard CT and recanalization success after MT remains controversial [34].

Thrombus perviousness was recently introduced as a new imaging biomarker for blood clot permeability estimation, describing contrast material penetration into the thrombus [18]. Perviousness is related to the permeability of the blood clot, which is closely linked to its physical structure and determines the amount of partial blood flow through the occluded artery [18,35,36]. It has been speculated that high permeability of the clot allows residual blood flow through the occluded artery, which may slow down tissue damage, increase the time window for MT, and correlates with MT outcome. In addition, thrombus permeability may increase the effect of a concomitant tissue-type plasminogen activator and also influence the efficacy of MT [37].

The measurement of thrombus perviousness in the clinical routine is challenging and, therefore, two simplified measures have been introduced: (1) the thrombus attenuation increase (TAI) and (2) the void fraction [18]. The thrombus attenuation increase is calculated as the mean clot density difference on NCCT and CTA [23,38]. In short, three spherical regions of interest (ROIs) with a diameter of 1–2 mm are manually placed on the clot, both on NCCT and CTA. The average of every three ROIs is calculated and used as *ρNCCT* and *ρCTA*, respectively. Perviousness is than computed as TAI = *ρCTA* − *ρNCCT*. The void fraction is calculated in a similar way with three ROIs placed on the thrombus in NCCT and CTA. The perviousness is the ∆_thrombus_ divided by ∆_blood_, where ∆_thrombus_ is the increase in attenuation of the thrombus between NCCT and CTA and ∆_blood_ is the increase in attenuation in the contralateral artery [18]. While the TAI is less accurate than the void fraction, it is accepted for determining clot perviousness in the clinical setting because it is faster to determine and the contralateral artery is not always fully visible due to oblique slice orientation.

Most clots will produce no or weak signals on NCCT (baseline), while their brightness on CTA is determined by how much contrast material they take up (high contrast uptake − high signal intensity). Perviousness is influenced by the clot main components: red blood cell, fibrin, and platelet. However, studies correlating perviousness to clot composition produced contradictory results. Several research groups found that pervious thrombi were associated with a higher percentage of red blood cells, whereas impervious clots had a higher fibrin/platelet content [15,39]. In contrast, others found permeable thrombi to contain a higher fibrin/platelet content and a lower percentage of red blood cells [22,38,40]. Yet another study observed no correlation between red blood cell and fibrin content and perviousness and suggested that pervious clots contained fewer platelets [23]. Several factors can explain these contradictory findings: Firstly, Berndt et al. [38], Patil et al. [22], and Hund et al. [39], (2020) used hematoxylin and eosin staining which cannot discriminate between fibrin and platelets and lumps them together in one fraction. One has to be aware of this limitation since fibrin and platelets have different biophysical properties. Secondly, composition is not the only factor influencing perviousness. Clot structure (e.g., outer shell, platelet-contraction) and the binding affinity of the components for contrast agent may also play a role. A high signal may be due to a loose clot (fast contrast uptake, fast washout) or due to one component strongly binding contrast agent (slow uptake, slow washout) [41].

Several studies reported that increased permeability was associated with better recanalization and/or better outcome [18,19,35,38]. However, some studies also reported contradictory results. Dutra et al. [19] observed an association between perviousness and outcome but none with recanalization. Kappelhof et al. [42] observed better response to thrombolytic treatment in patients with more pervious clots, but permeability had no effect on reperfusion success. Some studies even reported no correlation between perviousness and patient outcome nor Thrombolysis In Cerebral Infarction (TICI) scores [23].

The conflicting results of perviousness may be partly related to the short time between contrast material administration and the acquisition of CTA, not permitting sufficient time for the contrast to interact with occlusive thrombi [43]. Another reason may be linked to the density measurement methods, not taking into account the whole volume of the clot. To overcome these pitfalls, we used an additional late venous phase (CTV) time point after CTA and computed TAI between CTV and CTA to characterize late uptake and early washout of contrast agent, which we will call dynamic perviousness. Instead of characterizing perviousness using a two-dimensional (2D) spherical ROI covering only part of the clot volume, which is the gold standard of perviousness measure in the literature, TAI was computed voxel-wise for the entire visible 3D clot volume as an image matrix.

## 2. Methods

### 2.1. Patients

The study was approved by the regional ethical board. A retrospective analysis of 475 consecutive patients referred for MT due to LVO in our hospital between 2019 and 2022 was performed. Patients with occlusions of the intracranial internal carotid artery (ICA), proximal middle cerebral artery (MCA) up to the proximal M2 segment, and basilar artery were included. Further inclusion criteria were: (1) the availability of pre-intervention CT imaging (NCCT, CTA) with an in-plane resolution below 0.8 mm; (2) the availability of CTV after contrast agent administration; (3) relative low motion artifacts on CT images and (4) no previous contrast agent administration for another imaging procedure. This resulted in 137 patients, from which 65 patients with a visible hyperdense artery sign on NCCT were selected for further analysis. The NIHSS score at admission, revascularization results as measured by the modified TICI scale, and number of passes performed to achieve final recanalization were included in the analysis.

### 2.2. Imaging

CT was performed on a range of scanners from different vendors including Siemens Somatom X.cite, Somatom Definition Flash, Somatom Definition AS+, and Somatom Definition Edge Plus (Siemens, Erlangen, Germany), as well as GE Revolution (General Electric, Boston, MA, USA) and Philips Brilliance iCT 256 (Philips, Amsterdam, The Netherlands). The three-phase CT clinical protocol consists of NCCT, an arterial phase measured with CTA and CTV after intravenous contrast agent injection. Due to the different scanner types and acute setting of AIS, the CTV was timed with a mean delay of 70 ± 28 s after the CTA. A fixed tube voltage of 120 kV for both the unenhanced and venous phases was used.

### 2.3. Image Processing and Statistical Analysis

Postprocessing and statistical analysis were done with Python 3.16 (Python Software Foundation, Beaverton, OR, USA) and Matlab 2022 (The MathWorks, Inc., Natick, MA, USA). The postprocessing pipeline consisted of four steps: (1) image resampling and registration, (2) thrombus segmentation, (3) perviousness map computation and (4) statistical analysis of created segments.

**Figure 1:**
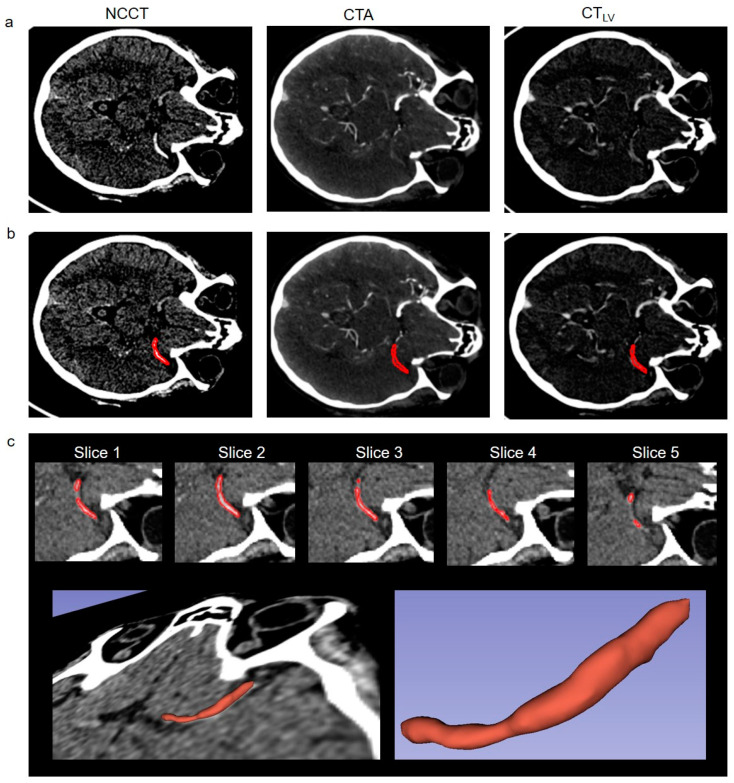
(**a**) Representative non-contrast CT (NCCT), CT angiography (CTA), and late venous phase CT (CTV) images of our clinical AIS protocol with corresponding segmentation. (**b**) Corresponding thrombus segmentations for the images in (**a**) are shown with red. (**c**) Zoomed-in NCCT images for each thrombus slice position and corresponding segmentation with red. Each 2D transversal slice for the visible clot volume was segmented in the 3D Slicer resulting in full 3D segmentation of the thrombus volume (**c**). Images show an ICA occlusion in a 70 year old patient with TICI III after intervention.

All images were resampled to a uniform 512 × 512 × 240 matrix size with 0.5 × 0.5 × 0.7 mm resolution and the CTA and CTV were co-registered to the NCCT volume using dipy (www.dipy.org, accessed on 12 February 2024) [44]. Thrombi were segmented manually by considering the hyperdense artery sign on NCCT. ROIs on 2D transversal NCCT slices were drawn manually using the free contour selection in 3D Slicer (www.slicer.org, accessed on 12 February 2024) [45], as illustrated in Figure 1. ROIs covered the entire visible thrombus section on NCCT (Figure 1b,c). ROIs of the clot were determined for each 2D transversal slice position on which the clot and its hyperdense artery sign was visible resulting in a fully 3D segmentation of the clot volume (Figure 1c). The defined ROIs on NCCT were applied on CTA and CTV.

Perviousness was analyzed at two time points: (1) between CTA and NCCT and (2) between CTV and CTA. TAI was computed voxel-wise in HU by subtracting from each other two corresponding images between the analyzed time points. This resulted in an image that characterized thrombus perviousness voxel-wise, which we call the perviousness map in this study. Three perviousness maps were computed: P_1_ between NCCT and CTA (P_1_ = CTA − NCCT), characterizing standard clot perviousness as used customarily in the literature; P_2_ between CTV and CTA, characterizing the late contrast uptake component (P_2_ = CTV − CTA); and P_3_ between CTA and CTV, characterizing the early contrast washout component (P_3_ = CTA − CTV). Voxels with negative values in P_1_, P_2_, and P_3_ were set to zero to contain information only about standard perviousness, late uptake, and early washout component of the clot, respectively.

Patients were divided into groups based on NIHSS at admission, TICI score, and number of MT passes. Mean HU in P_1_, P_2_, and P_3_ for the segmented thrombi volumes were computed and statistically analyzed for grouped parameters. In addition, the influence of the varying time delay between CTA and CTV was analyzed for the parameter groups. Significance analysis was done by the two-sided Wilcoxon–Mann–Whitney test in Matlab. For *p*-values less than 0.05, differences were considered as significant.

## 3. Results

**Figure 2:**
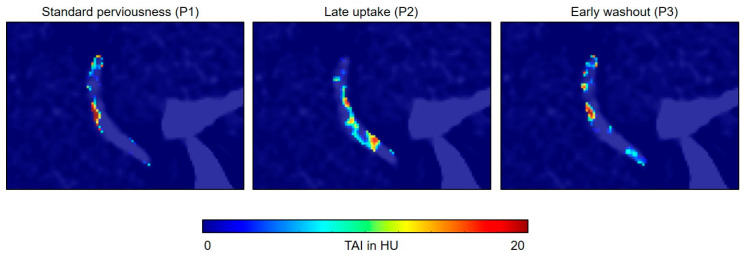
Example maps of standard perviousness (P1 = CTA − NCCT), late contrast uptake (P2 = CTV − CTA), and early contrast washout (P3 = CTA − CTV) for the clot shown in Figure 1 overlaid on the NCCT image. Voxels with negative values in P1, P2, and P3 were set to zero to contain information only about the standard perviousness, late uptake, and early washout components of the clot, respectively. Maps show sub-regional differences of the measured quantities for the clot volume. For example on P2, it is visually apparent that there is a thin area through the length of the clot in which contrast agent uptake increased after CTA while the rest of the clot showed no contrast agent uptake increase.

Representative images of our clinical AIS protocol in a 70-year-old male patient with an ICA occlusion are shown in Figure 1. The patient had a severe stroke with a Modified Rankin Scale (mRS) of 5. After reperfusion therapy with stent retriever and two passes, a complete reperfusion (TICI III) was achieved. Three months after the intervention, the patient had an mRS of 3. Figure 1c illustrates the definition of the ROIs, which were applied on NCCT, CTA, and CTV. Perviousness maps in Figure 2 show voxel-wise, the TAI between two corresponding imaging time points. It is visually apparent in the images that standard perviousness (P_1_), as well as the late uptake (P_2_), and early washout (P_3_) components of TAI show sub-regional differences for the entire clot volume (Figure 2). For example, on P_2_ in Figure 2, it is visually apparent that there is a thin area through the length of the shown example clot in which contrast agent uptake increased after CTA while the rest of the clot showed no increase in contrast agent uptake.

**Figure 3:**
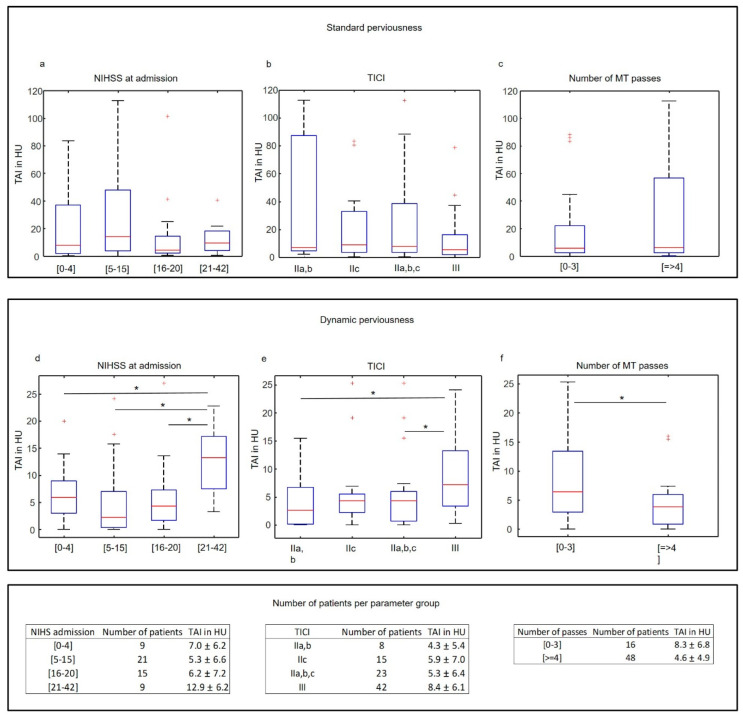
Statistical analysis of standard perviousness (**a**–**c**) and late contrast uptake (**d**–**f**) for NIHSS at admission, TICI, and the number of MT passes. Standard perviousness (**a**–**c**) is not associated with the analyzed parameters (*p* value of Wilcoxon–Mann–Whitney test is everywhere above 0.05). The mean late-uptake TAI of thrombi showed a significant correlation with the NIHSS score, TICI, and the number of MT passes (**d**–**f**). * in (**d**–**f**) indicates *p* values of Wilcoxon–Mann–Whitney test below 0.05. Outliers are marked with red crosses and were included in the statistical analysis.

**Figure 4:**
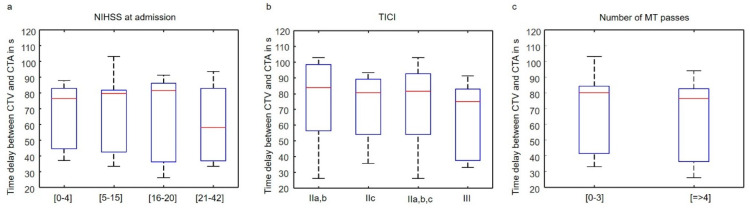
Statistical analysis of the time delay between CTA and CTV. No significant difference was measured between the parameter groups of NIHSS at admission (**a**), TICI (**b**), and the number of MT passes (**c**). *p*-values of the Wilcoxon–Mann–Whitney test are above 0.05 everywhere.

The late uptake component of dynamic perviousness correlated with initial NIHSS, the TICI score, and the number of MT passes. The mean late-uptake TAI of thrombi in patients with NIHSS scores greater than 21 at admission (indicating severe stroke) was significantly higher (ca. 100%) than in thrombi of patients with lower NIHSS scores (Figure 3d, 12.9 ± 6.2 vs. 6.2 ± 7.0 vs. 5.3 ± 6.6 vs. 7.0 ± 6.2 HU for NIHSS_21–42_, NIHSS_16–20_, NIHSS_5–15_ and NIHSS_0–4_, with *p* = 0.05, 0.005, and 0.01, respectively). Group mean late-uptake TAI of patients with TICI score III (indicating complete recanalization) was significantly higher (ca. 95%) than in patients with TICI score IIa and IIb (Figure 3e, 8.4 ± 6.1 vs. 4.3 ± 5.4 HU, *p* = 0.03). No significant difference was measured between TICI IIc and III, although the *p*-value was relatively small (Figure 3e, 8.4 ± 6.1 vs. 5.9 ± 7.0 HU, *p* = 0.05). Because of the small sample size of TICI IIa,b, (only 8 patients), we analyzed the group mean late-uptake TAI between TICI III and IIa,b,c, which was still significantly higher for TICI III (Figure 3e, 8.4 ± 6.1 vs. 5.3 ± 6.4 HU, *p* = 0.01). Group mean late-uptake TAI of thrombi requiring less than four MT passes was ca. 80% higher than for clots requiring four or more than four passes (Figure 3f, 8.3 ± 6.8 vs. 4.6 ± 4.9 HU, *p* = 0.03). No significant correlation between the mean standard perviousness (Figure 3a–c) as well as between the early washout component of thrombi and clinical parameters was found. The varying time delay between CTA and CTV did not differ significantly between the analyzed parameter groups, as shown in Figure 4.

## 4. Discussion

By introducing the concept of dynamic perviousness and voxel-wise characterization of the 3D segmented thrombus, we showed in this study that clots with late contrast uptake are significantly more prone to complete and quick revascularization, as compared to thrombi with different dynamic perviousness characteristics. Since the mean standard perviousness did not show any effect on the measured parameters, this finding underlines the potential role of the real-time dependent thrombus to contrast material interaction, i.e., dynamic perviousness measurement.

Thrombus properties and composition such as length [10,12,13,16,17], histological composition [10,16,17], stiffness [20], and perviousness [18,19,35,36,38] have all been associated with treatment success and clinical outcome. Several studies reported, that intervention times of MT for soft, red-blood-cell-rich clots, were shorter and a smaller number of removal attempts was required [11,27,46,47], while older, harder, and especially calcified clots were generally more difficult to remove [20,48,49,50,51]. MT techniques also influence success: aspiration was more successful with soft clots, whereas stent-retrievers were more successful with harder clots [10,11,52]. Although easier to remove, softer clots fragment or migrate more easily during MT, which can lead to distal embolisms and thus, prevent complete recanalization [17]. Understanding all these features with imaging and before intervention should help revascularization success and thus, patient outcomes.

The usefulness of thrombus perviousness for predicting clinical outcomes of MT has been intensively studied with conflicting results. Mishra et al. [36] found that pervious clots were associated with early reperfusion in patients treated with thrombolytic agents while Santos et al. [18,53] reported that higher thrombus perviousness was associated with improved functional outcomes, smaller final infarct volumes, and higher recanalization rates. Dutra et al. [19] observed an association between perviousness and better functional outcome but none with recanalization. In a study by Berndt et al. [38], perviousness showed an association with favorable outcomes and reperfusion. Similarly, Patel et al. [54] reported that perviousness correlated with a first-pass outcome in MT. In contrast to these studies, Mokin et al. [55] found an association between perviousness and first-pass angiographic success in patients treated with aspiration first approach for MT but perviousness did not significantly correlate with first-past success using a stent retriever. Similarly, Kappelhof et al. [42] observed better response to thrombolytic treatment in patients with more pervious clots, but permeability did not affect reperfusion success. Ye et al. [23] reported no correlation between thrombus density, perviousness, and reperfusion or clinical outcome and Byun et al. [56] found no correlation between perviousness and recanalization or successful revascularization in patients treated with a stent retriever.

The contradictory findings regarding the predictive value of clot perviousness in AIS may be attributed, at least partly, to the used evaluation methods. In all studies, mean perviousness was computed within spherical ROIs on a 2D transversal slice covering only part of the clot, which cannot depict the characteristics of the entire clot volume. In addition, perviousness was computed based only on NCCT and CTA, neglecting the late-phase contrast uptake of thrombi, which may have an important relationship with thrombus mechanical parameters and revascularization. Therefore, we used a full 3D segmentation combined with voxel-wise characterization of dynamic perviousness, including a late venous phase time point after CTA.

Using 3D volumetric evaluation and voxel-wise perviousness mapping, we did not measure any significant correlation between mean standard perviousness and TICI score or number of MT passes. This finding is in agreement with Ye et al. and Byun et al. and strongly suggests that standard perviousness computed on arterial phase CTA is not as strongly correlated with recanalization success as previously reported [18,35,36,38,53,54].

In contrast to standard perviousness, the late uptake component of contrast agent penetration into the thrombus computed in the late venous phase after CTA was significantly correlated both with the TICI score as well as with the number of MT passes (Figure 3). The only two other publications focusing on more than one phase of time-dependent perviousness reported contradictory results. Santos et al. [53] computed dynamic perviousness using the arterial, venous, and delayed phases of CTA and found that perviousness on arterial phase CTA was superior to venous phase CTA. In contrast, Chen et al. [57] computed the time-resolved curve of contrast agent uptake using multiple time points from CT perfusion and a relatively large time resolution. They reported that thrombus permeability assessed on dynamic CTA was a better biomarker for predicting good outcomes in patients with AIS than perviousness on single-phase CTA. Our findings are in line with Cheng et al. and suggest that the late uptake component of contrast agent uptake should be considered in perviousness analysis.

It is thought that contrast agent uptake by the thrombus is a dynamic process that is significantly influenced by several physical factors, such as thrombus shape, density, stiffness, and porosity. Red-blood-cell-dominated clots are less stiff and are more deformable than clots with a high platelet ratio [20,58,59,60,61], while platelet-driven forces during thrombus maturation can compact red blood cells into impermeable layers of polyhedrocytes, which leads to a higher overall stiffness and decreased porosity [62,63]. It has been shown that the red blood cell/fibrin ratio and corresponding clot density are strongly associated with contrast agent uptake [40]. Our findings suggest that standard thrombus perviousness is suboptimal for the prediction of revascularization outcome, which can be better addressed by the concept of dynamic perviousness, which allows more time for contrast-to-clot interaction.

Although we obtained encouraging results, our study has some limitations. First, because of its retrospective design, the CT protocols did not use a constant time delay between CTA and CTV. In the acute treatment of AIS, it is a challenge to maintain precise time intervals between CT acquisitions. To test its influence on the statistical analysis, the time delay between CTA and CTV was analyzed for the parameter groups and no significant differences were measured (Figure 4). Nevertheless, the varying time delay between CTA and CTV introduces a potential bias into our analysis. Second, because we applied 3D segmentation, only patients with a visible hyperdense artery sign on NCCT were included in the analysis and our findings are therefore valid only for this patient group. Third, the number of patients in the analyzed parameter groups was unbalanced. We had only eight patients with TICI IIa,b, which were statistically compared to a much larger group of 40 patients with TICI III. Therefore, we analyzed the union of TICI IIa,b and c against TICI III and the difference was still statistically significant, indicating that thrombi with complete reperfusion (TICI III) have different imaging characteristics on late uptake maps than lower-scored thrombi. In the future, it would be of great interest to apply the 3D image analysis of the late uptake component of thrombi presented here to a larger database of AIS patients.

## 5. Summary

We presented here a 3D volumetric evaluation of dynamic perviousness in thrombi in AIS. Our results suggest that the late uptake component of contrast agent uptake in thrombi may play an important role in estimating revascularization parameters using pre-interventional CT, whereas the state-of-the-art measure of thrombus perviousness on single-phase CTA is not as strongly correlated with recanalization success as previously reported.

## Data Availability

Data is available upon reasonable request.

## Abbreviations

AIS, acute ischemic stroke; CT, computed tomography; NCCT, non-contrast CT; CTA, CT angiography; CTV, late venous phase CT; HU, Hounsfield units; MT, mechanical thrombectomy; TAI, thrombus attenuation increase; NIHSS, NIH Stroke Scale scores; TICI, Thrombolysis In Cerebral Infarction; ICA, intracranial internal carotid artery; MCA, proximal middle cerebral artery; ROI, region of interest.
